# A Novel Squid Ink–Quinolin Derived Compound (AEBI), Promotes Hair Growth and Enhances Hair Follicle Stem Cell Activity

**DOI:** 10.3390/ijms27209039

**Published:** 2026-10-12

**Authors:** Xiaoqing Tian, Weiqi He, Youshiqi Zhou, Xiaozhen Diao, Chengqi Fan, Yanan Lu, Fenghua Tang, Na Li, Wenhui Wu

**Affiliations:** 1 Department of Marine Pharmacology, College of Food Science and Technology, Shanghai Ocean University, Shanghai 201306, China; amytian0904@126.com (X.T.); 15962655586@163.com (W.H.); youshiqi0403@163.com (Y.Z.); xzdiao@shou.edu.cn (X.D.); luyn@ecsf.ac.cn (Y.L.); 2 Key Laboratory of Oceanic and Polar Fisheries, East China Sea Fisheries Research Institute, Chinese Academy of Fishery Sciences, Ministry of Agriculture and Rural Affairs, Shanghai 200090, China; fancq@ecsf.ac.cn (C.F.); f-h-tang@163.com (F.T.); 3 Putuo Branch of International Combined Research Center for Marine Biological Sciences, Zhoushan 316104, China; 4 Marine Biomedical Science and Technology Innovation Platform of Lin-Gang Special Area, Shanghai 201306, China

**Keywords:** squid ink, AEBI, HFSCs, rat, hair growth

## Abstract

Squid ink is a marine-derived bioresource containing diverse bioactive compounds; however, the biological functions of its low-molecular-weight constituents remain largely unexplored. In this study, a novel quinolin derivative, named AEBI, was isolated and identified from squid ink through enzymatic hydrolysis, chromatographic purification, and spectroscopic analysis. The hair growth–promoting activity of AEBI was subsequently evaluated using mouse hair follicle stem cells (HFSCs) and a depilation model established in male Sprague–Dawley (SD) rats. In vitro, AEBI significantly enhanced HFSC viability in a concentration-dependent manner and increased the expression of stem cell- and extracellular matrix-associated proteins, including p63, laminin, and fibronectin. In vivo, topical administration of AEBI markedly accelerated hair regrowth, increased hair follicle density, promoted follicular maturation, and improved hair shaft morphology in depilated rats. Histological analysis revealed that AEBI treatment increased the proportion of mature hair follicles and enhanced subcutaneous adipose tissue development. Furthermore, AEBI treatment upregulated the expression of hair growth–related molecules, including Wnt10b, β-catenin, LEF1, and Ki-67, suggesting that modulation of Wnt/β-catenin-associated signaling may contribute to its hair growth–promoting effects. Notably, high-dose AEBI exhibited comparable or superior effects to 5% minoxidil in several evaluated parameters. Collectively, these findings identify AEBI as a novel squid ink–derived bioactive molecule with potential applications in the development of natural hair growth–promoting agents and provide new insights into the utilization of marine-derived small molecules.

## 1. Introduction

Squid ink is a natural defensive substance secreted by cephalopods such as squids, octopuses, and cuttlefish, and it is abundant in diverse species [1,2,3]. As early as the Tang Dynasty, the renowned pharmacologist Chen Cangqi recorded the medicinal value of cuttlefish ink in “*Supplement to the Materia Medica*”, documenting its efficacy in treating “blood stasis and cardiac pain” [4]. Squid ink contains a variety of functional ingredients, including melanin, polysaccharides, proteins, lipids, and multiple minerals. Existing studies have shown that squid ink polysaccharide (SIP), with a molecular weight of 40–100 kDa, exhibits multiple biological activities, including neuroprotective, antioxidant, anti-inflammatory, anti-tumor, and antibacterial effects, which confer beneficial effects on human health [5,6,7,8]. A novel squid ink–derived protein, lolduvin-S (60 kDa), exhibits potent L-amino acid oxidase activity [9]. Additionally, protein and peptide complexes derived from squid ink possess antibacterial, anti-tumor, and surimi gel-enhancing capacities, allowing their extensive application as functional food additives [1,2,3,5]. Current studies on squid ink mainly concentrate on melanin and its derivatives. The major melanin constituent in squid ink is eumelanin, a high-molecular-weight polymer biosynthesized by oxidative polymerization of tyrosine, and composed of 5,6-dihydroxyindole (DHI) and 5,6-dihydroxyindole-2-carboxylic acid (DHICA). It has a variable molecular weight and presents a dark brown appearance [1,10]. Eumelanin possesses diverse biological activities, including anti-tumor, anti-allergy, antioxidant, and free radical scavenging activities [11,12,13]. However, the isolation and bioactivity exploration of small-molecule compounds from squid ink remain insufficiently investigated.

However, the isolation and bioactivity exploration of small-molecule compounds from squid ink remain insufficiently investigated. Therefore, the identification and characterization of novel low-molecular-weight bioactive compounds from squid ink may provide new opportunities for the development and utilization of marine-derived bioresources.

In the present study, a novel quinoline derivative, named AEBI, was isolated and identified from squid ink. Its potential hair growth–promoting activity was evaluated using mouse hair follicle stem cells (HFSCs) and an in vivo rat depilation model. HFSCs were used to investigate the effects of AEBI on cellular viability and the expression of stem cell- and extracellular matrix-associated proteins, whereas the rat depilation model was used to evaluate its effects on hair regrowth and hair follicle development in vivo. This study provides new insights into the biological activities and potential applications of low-molecular-weight compounds derived from squid ink.

## 2. Results

### 2.1. The Screening of Active Molecules from Crude Squid Ink Extract

The supernatant derived from enzymatic hydrolysis of the original sample was collected after protein removal via chloroform extraction. The aqueous phase was further purified using Sephadex-G25 gel column chromatography to yield three fractions: YY-I, YY-II, and YY-III. These fractions were subsequently ultrafiltered using a 5 kDa ultrafiltration membrane to acquire small-molecule fraction 1 (Y2 < 5KD). After successive purification procedures, the experimental samples were fully prepared for subsequent experimental assays.

**Figure 1:**
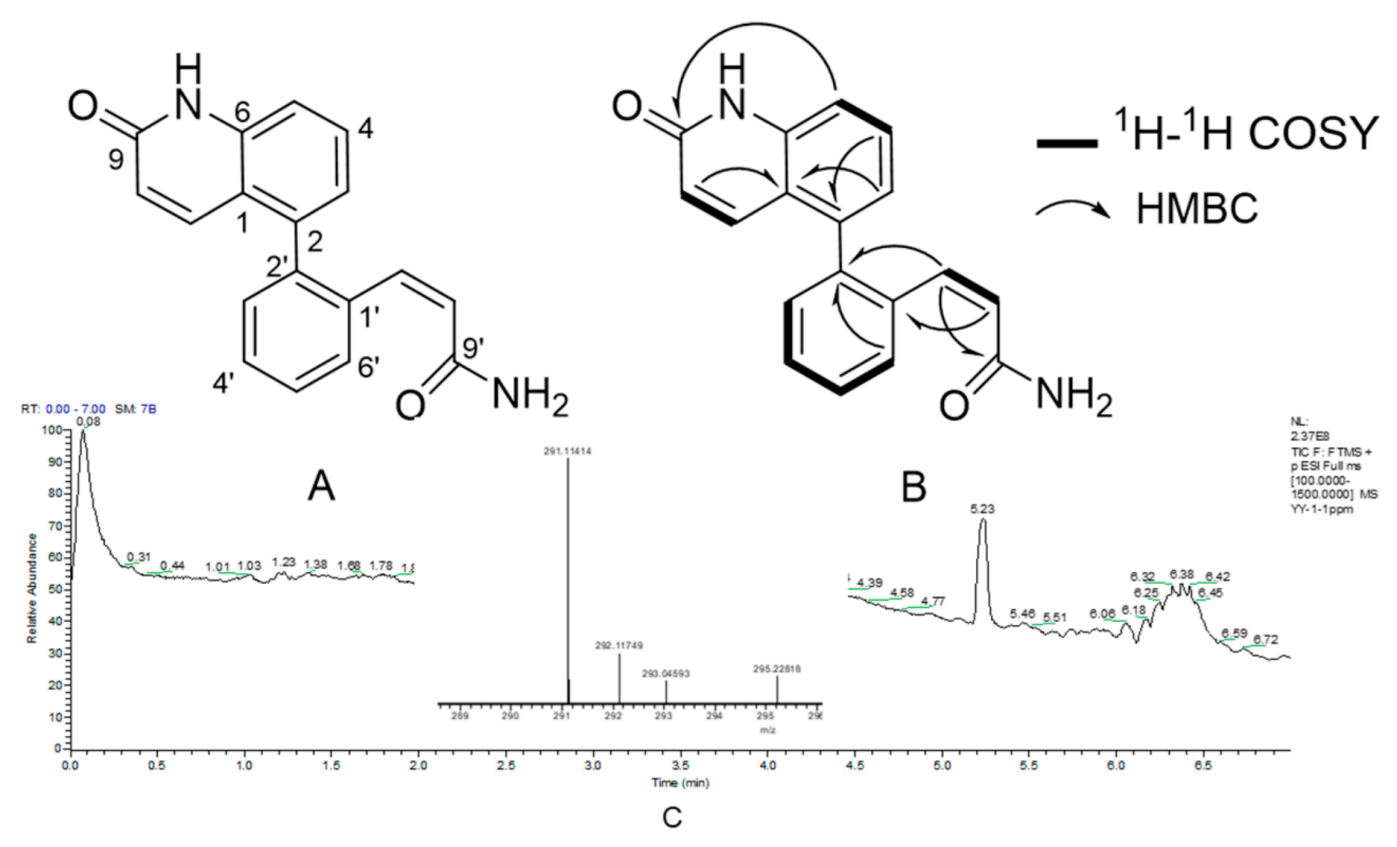
The active molecule was isolated from fraction 1, and its structure was determined to be quinolin (AEBI). (**A**) The structure of AEBI, (**B**) key HMBC(H → C) and COSY(H → H) correlations of AEBI, (**C**) HR-ESI-MS spectrum and purity chart.

Semi-preparative high-performance liquid chromatography (HPLC) analysis revealed that *Sthenoteuthis oualaniensis* cuttlefish ink is rich in diverse secondary metabolites. Peaks with favorable peak symmetry and distinct ultraviolet absorption were selected for preparative isolation. After fraction pooling and collection, a novel quinoline derivative was successfully isolated from the cuttlefish ink and named AEBI. Its structure was elucidated by NMR and HRESIMS, as shown in Figure 1 and Table 1. Its molecular formula of C_18_H_14_N_2_O_2_ was determined by the associated HRESIMS data (*m*/*z* [M+H]^+^ 291.1141, calcd for C_18_H_15_N_2_O_2_, 291.1128). AEBIIt’s IUPAC name is (Z)-3-(2-(2-oxo-1,2-dihydroquinolin-5-yl)phenyl)acrylamide. The corresponding spectra are provided in the Supplementary Material (Figure S1).

### 2.2. AEBI Improves Generation of HFSCs In Vitro

**Figure 2:**
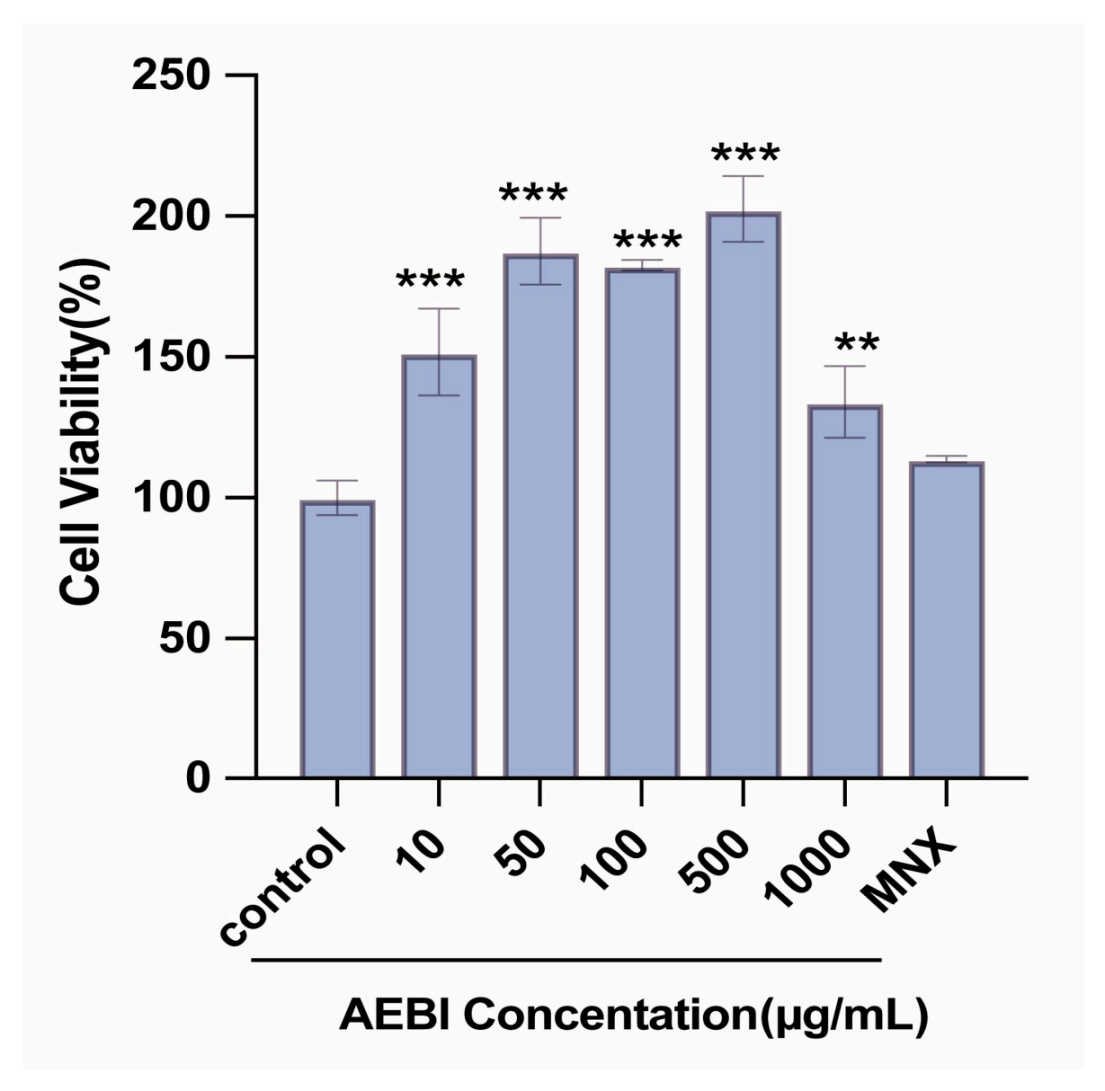
The cell viability changes of hair follicle stem cells (HFSCs) after co-culturing with extract 1 at different concentrations for 8 h are shown. In this experiment, the CCK-8 kit was used to detect the cell viability of HFSCs after treatment with samples corresponding to various concentrations. The results are expressed as the mean ± standard deviation of three independent experiments. (Compared with the control group, ** *p* < 0.01, *** *p* < 0.001).

Figure 2 illustrates the changes in cell viability of hair follicle stem cells (HFSCs) following 8-h incubation with different concentrations of extract 1. In this study, a CCK-8 assay kit was used to evaluate the cell viability of HFSCs after treatment with serially diluted samples. The results in Figure 2 reveal that AEBI exerts the strongest promotive effect on HFSC viability. HFSCs viability showed a markedly increasing trend across different concentration gradients and rose significantly with elevated treatment concentrations, reaching nearly 200% at the concentration of 500 μg/mL. Notably, all AEBI concentration groups exhibited higher cell viability than the positive control (MNX) group, verifying its superior proliferative activity compared with the positive control.

Taken together, AEBI is the most effective sample for improving HFSCs viability among all tested samples. It possesses prominent concentration-dependent promotive effects and shows superior application potential relative to the positive control.

### 2.3. AEBI Upregulates p63, Laminin, Fibronectin and TGF-β Expression in HFSCs

After 8 h of treatment of HFSCs with AEBI at different concentrations, ELISA detection results of AEBI confirmed that AEBI exerted positive regulatory effects on the expression of four key proteins, including p63, laminin, fibronectin, and TGF-β. Moreover, such regulatory effects displayed an obvious concentration-dependent trend.

**Figure 3:**
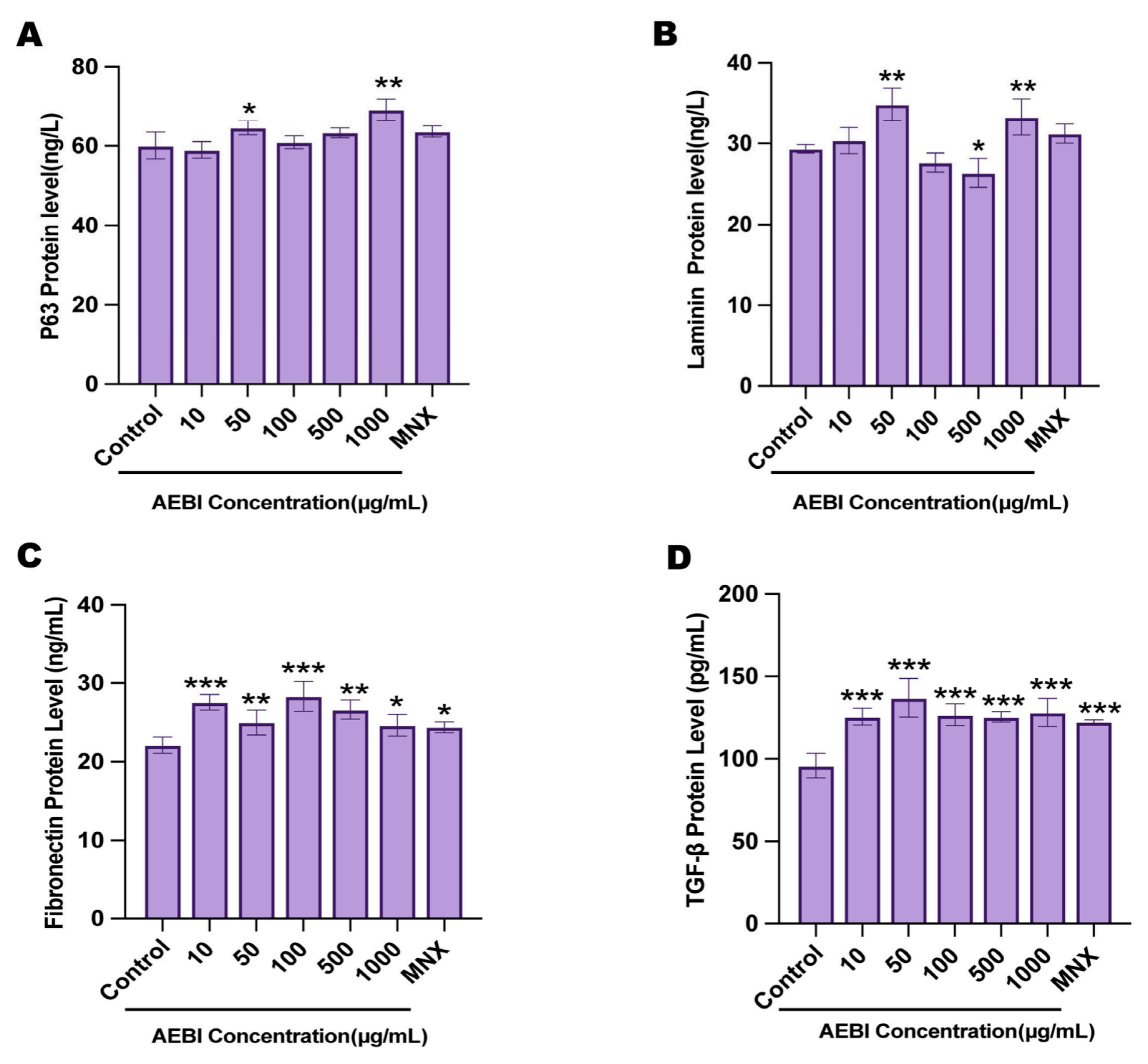
Presents the expression levels of four target proteins in HFSCs after 8-h treatment with AEBI at concentrations of 10, 50, 100, 500, and 1000 μg/mL, as determined by ELISA. (**A**) p63 expression level; (**B**) laminin expression level; (**C**) fibronectin expression level; (**D**) TGF-β expression level. All data are presented as the mean ± standard deviation of three independent experiments (* *p* < 0.05, ** *p* < 0.01, *** *p* < 0.001 vs. the control group).

Figure 3A shows that the regulatory effect of AEBI presents a concentration-dependent upward trend. As the treatment concentration increases, the expression level of P63 protein gradually rises, with a markedly elevated level detected in the 1000 μg/mL concentration group. The expression level of P63 in the positive control MNX group exhibits no statistically significant difference from the blank control group, which demonstrates the superior regulatory capacity of AEBI on the stemness maintenance of HFSCs.

Figure 3B reveals that AEBI exerts concentration-selective activation of laminin protein expression. Laminin levels in the 50 μg/mL and 1000 μg/mL groups are highly significantly different from those of the control group. At 50 μg/mL, laminin expression reaches its maximum. Laminin abundance in the MNX control group remains comparable to the blank control, indicating that AEBI achieves more stable activation of extracellular matrix (ECM) modulation in the hair follicle basement membrane.

Regarding fibronectin protein (Figure 3C), the effective concentration coverage range of AEBI’s regulatory effect is broader, with significant upregulation of protein expression levels observed in most gradient groups within the concentration range of 10~1000 μg/mL. At 10 μg/mL and 100 μg/mL, the expression levels of fibronectin were 27.58 ± 0.99 pg/mL and 28.32 ± 1.89 pg/mL, respectively. This finding underscores its broad-spectrum regulatory potential for adhesion and matrix anchoring functions in the HFSCs microenvironment.

Figure 3D indicates that all AEBI treatment groups markedly upregulate TGF-β protein expression, and such elevation remains steady and pronounced across tested concentrations. The peak expression level is observed at the concentration of 50 μg/mL.

### 2.4. AEBI Promotes the Expression of Hair-Related Proteins

**Figure 4:**
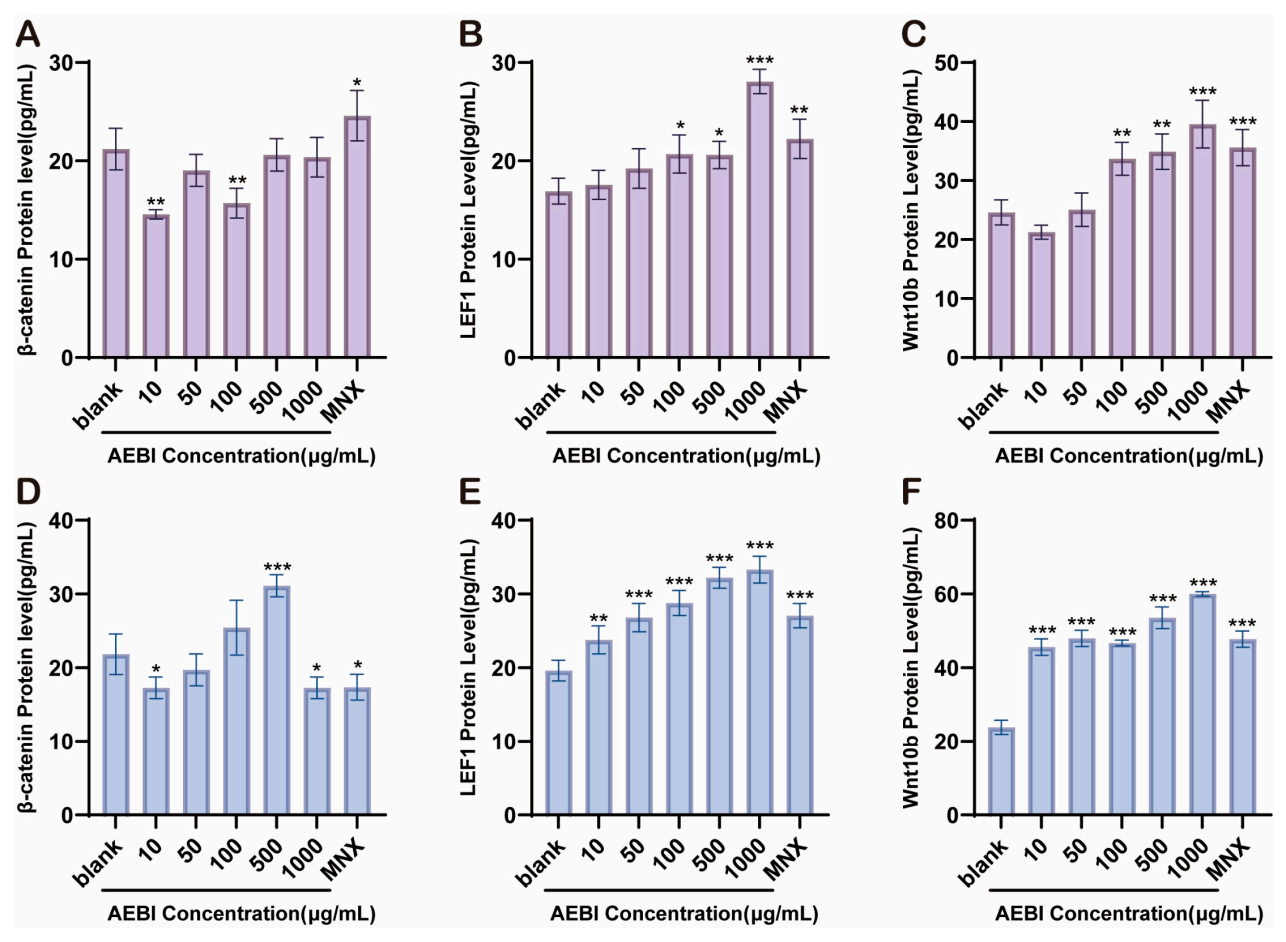
The expression levels of β-catenin, LEF1, and Wnt10b proteins in the dorsal skin after treatment with various AEBI concentrations, measured by enzyme-linked immunosorbent assay (ELISA). Panels (**A**–**C**) show data on day 7 for (**A**) β-catenin, (**B**) LEF1, and (**C**) Wnt10b, respectively. Panels (**D**–**F**) show data on day 14 for (**D**) β-catenin, (**E**) LEF1, and (**F**) Wnt10b, respectively. The group treated with 0 mg/mL AEBI served as the blank control, and 5% minoxidil was set as the positive control. All data are expressed as mean ± standard deviation of three independent experiments. (* *p* < 0.05, ** *p* < 0.01, *** *p* < 0.001, vs. blank control group).

The core components of the Wnt signaling pathway, including LEF1, β-catenin, and Wnt10b, were examined under different concentrations and incubation durations. The results are shown in Figure 4A–C.

Figure 4A demonstrates an obvious time- and concentration-dependent upregulation trend. Within the concentration range of 10~1000 μg/mL, LEF1 expression in the 14-day treatment group was significantly higher than that in the 7-day group, and it increased gradually as the treatment concentration rose. LEF1 expression reached the peak value of 33.30 ± 1.82 pg/mL. LEF1 levels in all treatment groups were markedly higher than those in the blank control group. LEF1 expression remained low and stable in the MNX control group and in the 100–1000 μg/mL high-concentration treatment groups on Day 14. These results confirm that AEBI promotes LEF1 protein expression in rat hair follicle stem cells. Figure 4B shows a similar changing trend. At most concentrations, β-catenin expression in the 14-day treatment group was higher than that in the 7-day group. Protein expression increased with the rise of treatment concentration, and significant differences existed between the 50~1000 μg/mL groups and the blank control group [14]. β-catenin content stayed relatively stable in the MNX control group and was lower than that in the 1000 μg/mL treatment group. As illustrated in Figure 4C, Wnt10b levels at all effective concentrations in the 14-day group were significantly higher than those in the 7-day group [15]. A distinct dose-dependent upward trend was observed at concentrations ranging from 10 to 1000 μg/mL, and all treatment groups had higher Wnt10b expression than the blank control group. Wnt10b expression in the MNX control group was lower compared with the 500–1000 μg/mL treatment groups [16,17,18].

### 2.5. AEBI Promotes the Growth of Hair on the Back of Rats

**Figure 5:**
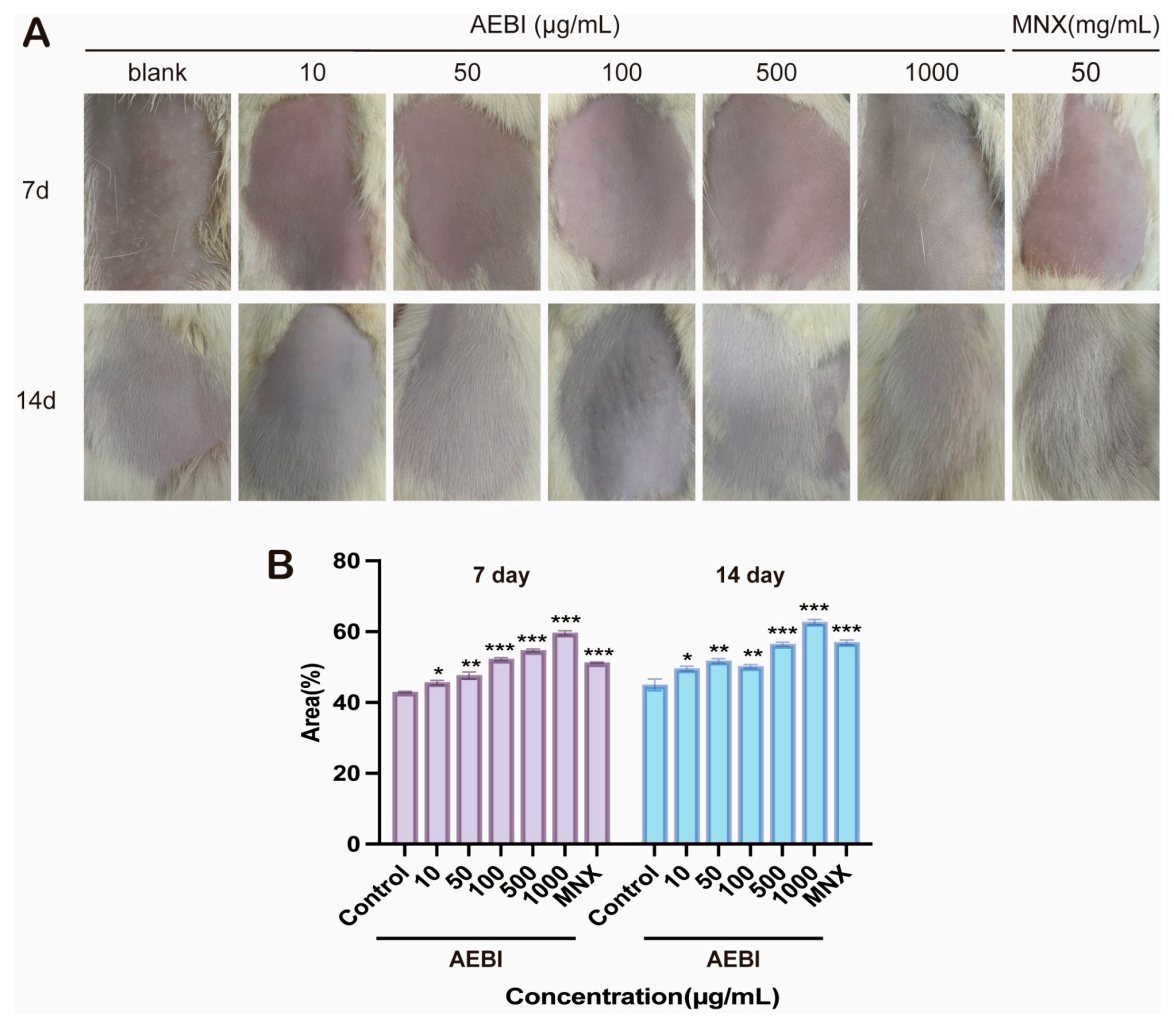
Treat the back skin of rats with AEBI at different concentrations, administering the treatment once every 24 h. Hair growth changes were observed daily, and photographs were taken on day 7 and day 14 (**A**). Hair coverage was quantified by pixel-based image analysis using ImageJ (version 1.54a; Java 1.8.0_112, 64-bit, National Institutes of Health, USA) on standardized 100 × 100 pixel regions of interest cropped from each photograph; the percentage of hair coverage per region is presented in (**B**). All data are expressed as mean ± SD. (* *p* < 0.05, ** *p* < 0.01, *** *p* < 0.001, vs. blank control group).

Figure 5A,B illustrate rat dorsal skin after 7 and 14 days of treatment with different concentrations of AEBI, with MNX treatment serving as the positive control. Over the 7–14-day treatment period, increased AEBI concentrations (10–1000 μg/mL) led to a gradual elevation in hair density and coverage within the depilated area; the 14-day treatment group exhibited superior overall hair coverage compared with the 7-day group. Quantitative analysis of hair coverage proportion (Figure 5B) showed that in the 7-day treatment cohort, hair coverage increased with rising AEBI doses, and most AEBI groups displayed statistically significant differences relative to the blank control group.

### 2.6. AEBI Increased the Number of Hair Follicles

**Figure 6:**
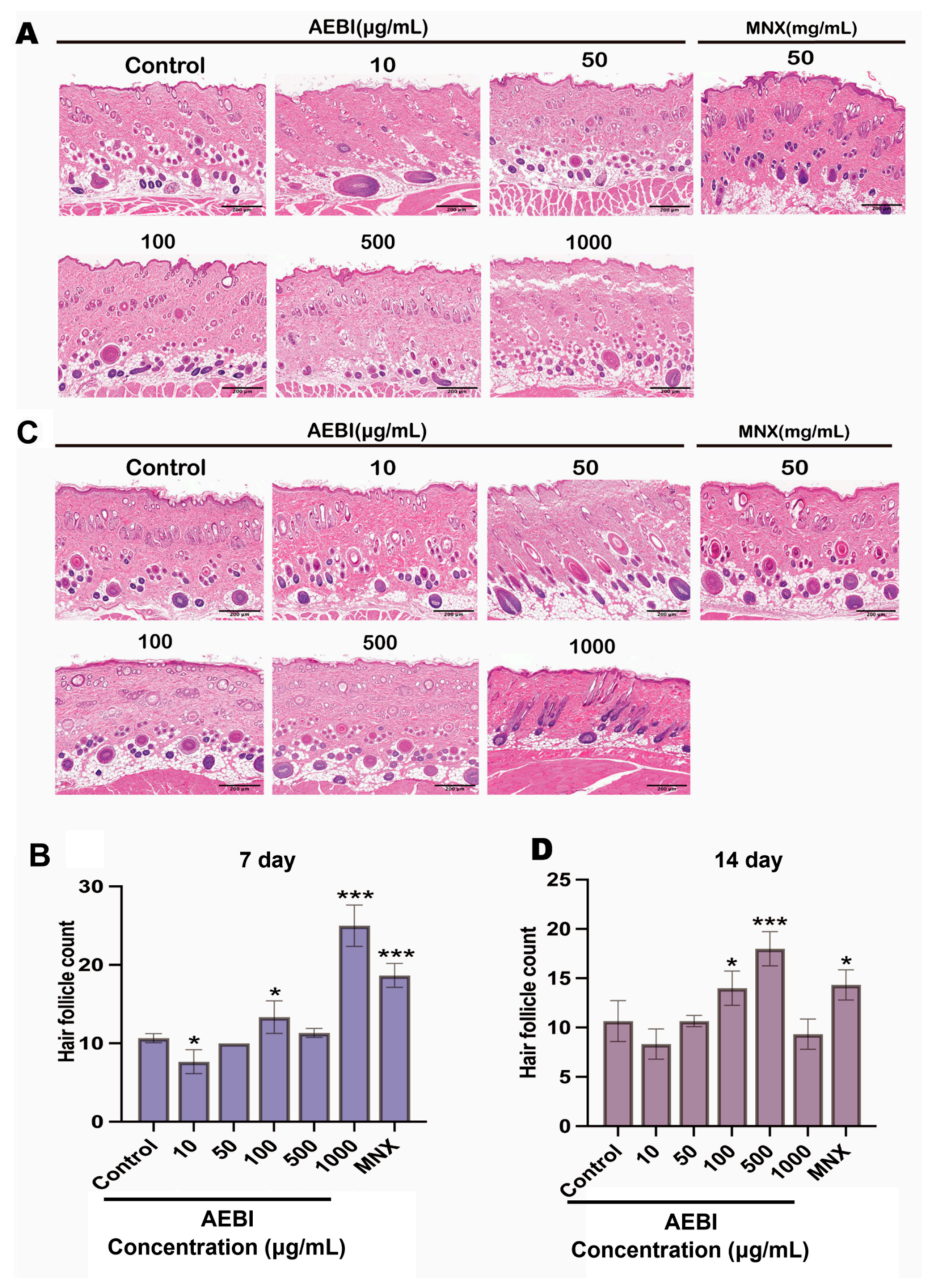
Illustrates hair growth variations in terms of follicle morphology and quantity after AEBI intervention, with 5% minoxidil and 0 mg/mL blank control set as references. (**A**,**B**) Representative longitudinal and transverse H&E-stained skin tissue sections on Day 7 (**A**) and Day 14 (**B**). Scale bar: 200 μm. (**C**,**D**) Statistical quantification of hair follicle numbers in each group, with (**C**) corresponding to Day 7 and (**D**) to Day 14. All data are expressed as mean ± standard deviation of three independent experiments. (* *p* < 0.05, *** *p* < 0.001 vs. blank control group).

Rats were topically administered AEBI at various concentrations on dorsal skin for 14 consecutive days. Obvious hair regeneration was observed on Day 7, and hair growth in depilated regions was further improved on Day 14, accompanied by elevated hair follicle maturity. Hematoxylin and eosin (H&E) staining results revealed that hair follicles in all AEBI-treated groups stayed in the anagen phase. Compared with the blank control and 5% minoxidil (MNX) positive control groups, these follicles presented higher density, intact morphology, larger quantity, and a thicker subcutaneous fat layer [17,19,20]. On Day 7, hair follicle count and maturity in the 500 μg/mL and 1000 μg/mL AEBI groups were markedly better than those in the MNX group (Figure 6A,C).

Hair follicle quantity was quantified by analyzing three high-power visual fields of H&E-stained slices. As shown in Figure 6B,D, hair follicle number exhibited a dose-dependent elevation in groups treated with AEBI at concentrations ≥ 10 μg/mL. On Day 7, the 1000 μg/mL group obtained a significantly higher follicle count of 25.00 ± 2.65. On Day 14, follicle number in the 500 μg/mL group rose remarkably to 18.00 ± 1.73. Additionally, AEBI treatment induced obvious thickening of the subcutaneous fat layer relative to both control groups. The above findings verify that AEBI can effectively facilitate hair growth. High-dose AEBI groups exerted comparable or even superior effects on hair follicle regeneration relative to MNX, suggesting promising application prospects in hair growth promotion.

### 2.7. Increased Number of Hair Follicle Proteins

The molecular mechanism underlying hair follicle regeneration constitutes a sophisticated process modulated by multiple stages and regulatory factors. The Wnt/β-catenin signaling pathway acts as a core regulator during hair follicle regeneration, and it is the earliest identified pathway governing basal plate and hair follicle development initiation.

**Figure 7:**
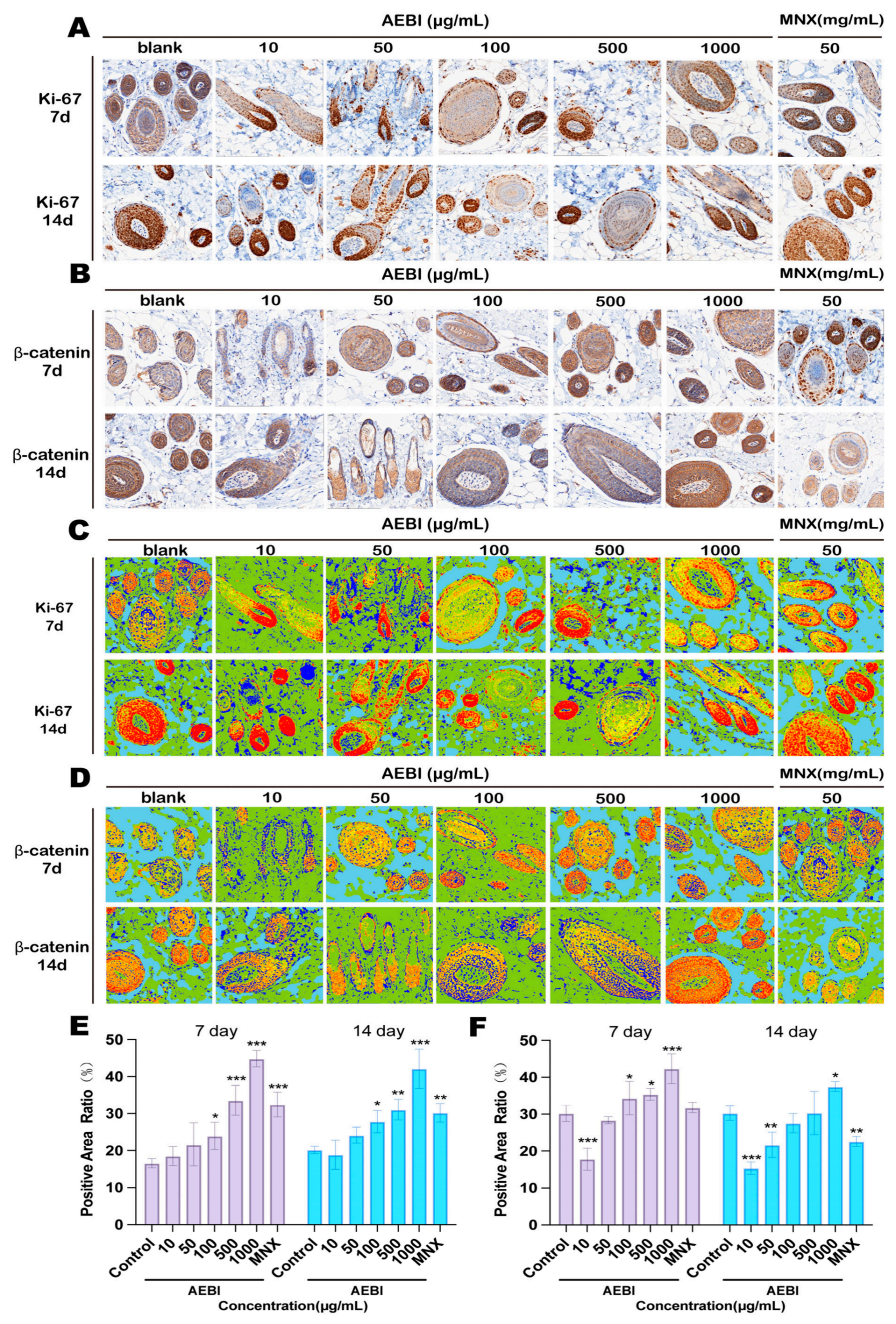
(**A**,**B**) Original immunohistochemical (IHC) staining images of Ki-67 (**A**) and β-catenin (**B**); Scale bar: 50 μm; Positive staining presents brownish-yellow signals. (**C**,**D**) Pseudocolor-converted images of Ki-67 (**C**) and β-catenin (**D**). Images were processed via the same image analysis software used for statistical quantification. Red regions indicate positive staining areas, and blue regions correspond to tissue background. (**E**,**F**) Bar charts showing the positive area percentage of Ki-67 (**E**) and β-catenin (**F**); Data were calculated from pseudocolor images using identical analytical software. Calculation formula: positive area percentage = (positive staining area/total visual field area) × 100%. The y-axis denotes positive area percentage (%). Error bars represent mean ± standard deviation (mean ± SD). Statistical comparison: * *p* < 0.05, ** *p* < 0.01, *** *p* < 0.001 versus blank control group. n = 3. Three densely distributed hair follicle visual fields (20× magnification) were randomly selected from each group for quantitative analysis.

To further elucidate the molecular mechanism whereby AEBI facilitates hair follicle regeneration, immunohistochemical staining was adopted to detect the expression of Ki-67 and β-catenin. Ki-67 serves as a proliferation biomarker that reflects cellular proliferative activity, while β-catenin functions as a pivotal effector of the Wnt pathway to modulate the hair follicle growth cycle. Tissue samples were collected and analyzed at 7 and 14 days post-treatment, respectively (Figure 7).

Immunohistochemical analysis identified brownish-yellow granular staining in the cytoplasmor nucleusas positive signals, whereas no specific staining was observed in negative control samples. Ki-67 (Figure 7A,C): Weak Ki-67 immunoreactivity was detected in the blank control group on both Day 7 and day 14, predominantly scattered in hair follicle stromal cells with a low positive ratio. With rising AEBI concentrations, Ki-67 signal intensity and positive cell distribution range gradually increased, primarily localized to the proliferative region of hair follicle epithelium. At identical doses, the 14-day group exhibited stronger overall signals than the 7-day group. Notably, 500 μg/mL and 1000 μg/mL AEBI induced moderate positive staining on Day 7 and intense staining on Day 14. The Ki-67 positive rate was higher in high-dose AEBI groups (100–1000 μg/mL) than in the MNX group (Figure 7E), with extremely significant differences relative to the blank control group [15].

β-catenin (Figure 7B,D): In blank control tissues, β-catenin positive signals were mainly distributed on the hair follicle cell membrane, presenting faint staining and limited positive coverage. AEBI treatment induced concentration-dependent signal enhancement and gradual expansion of positive staining areas. On Day 7, obvious cytoplasmic and nuclear positive staining was seen in groups treated with AEBI ≥ 100 μg/mL, whose positive areas were markedly larger than those of blank controls. On Day 14, signals further accumulated. High-concentration groups (500, 1000 μg/mL) displayed positive staining covering the entire hair follicle proliferative zone, with significantly deeper staining intensity compared with the 7-day counterparts at equal concentrations. MNX group showed lower β-catenin signal intensity, nuclear translocation level, and positive coverage than the high-dose AEBI groups. Statistical results (Figure 7F) revealed that β-catenin positive area percentages in 100–1000 μg/mL were significantly elevated. AEBI groups were significantly elevated versus blank. At the same concentration, positive area expansion was more pronounced on Day 14 than on Day 7 [14].

To ensure the objectivity of the results, standardized quantitative analysis was performed on the positive signals: three dense fields of hair follicles were randomly selected for each sample under a 20 × microscope, and image analysis software consistent with the pseudocolor image generation was used to directly calculate the proportion of positive area (positive staining area/total field of view × 100%) [21] (Figure 7C,D show Ki-67 and β-catenin pseudocolor images, with red areas indicating the positive staining area, providing visual support for quantitative analysis. The statistical analysis showed that the proportions of Ki-67- and β-catenin-positive areas were significantly higher in the AEBI-treated groups at various concentrations than in the blank control group, with generally higher levels observed on Day 14 than on Day 7 (Figure 7E,F). These results demonstrate that AEBI treatment was associated with increased Ki-67 and β-catenin expression in hair follicles [15,16,18,20].

### 2.8. AEBI Enhances Hair Diameter and Improves the Smoothness of Hair Cuticles

**Figure 8:**
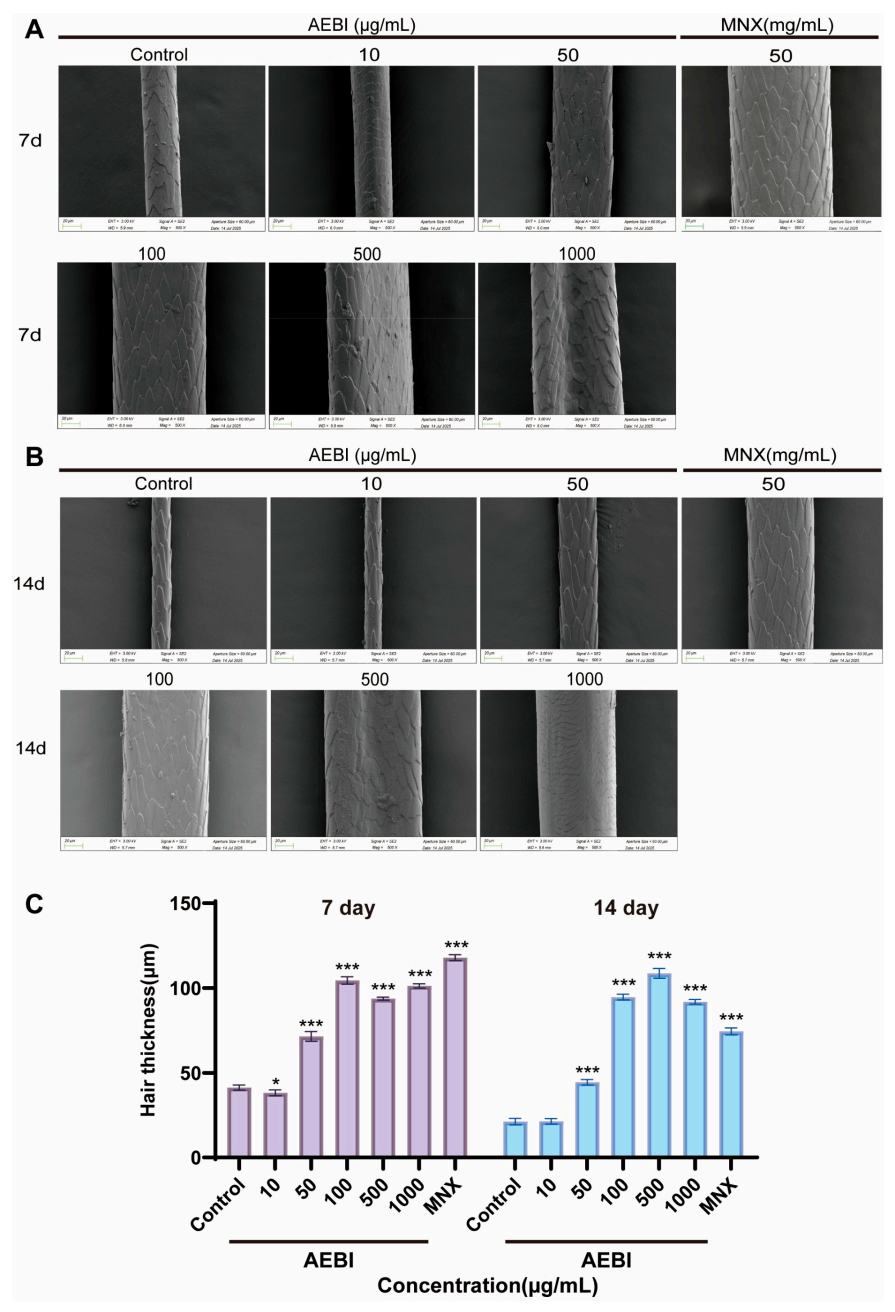
SEM images of rat hair from different treatment groups. (**A**,**B**) Representative SEM micrographs of hair samples on day 7 and day 14. (Scale bar: 20 µm). (**C**) Bar graphs of quantitative statistical analysis of hair diameter in each group on day 7 and Day 14. All data are presented as the mean ± standard deviation from three independent experiments. (* *p* < 0.05, *** *p* < 0.001 vs. blank control group).

Combining the scanning electron microscopy (SEM) morphological observations in Figure 8A,B with the quantitative statistical data on hair diameter in Figure 8C, the following conclusions were drawn. SEM observation of hair microstructure demonstrated that AEBI treatment was associated with changes in hair cuticle morphology depending on AEBI concentration and treatment duration. On Day 7 of treatment, hair cuticles became progressively more regular and fuller with increasing AEBI concentrations (10–1000 μg/mL). After 14 days of treatment, the hair cuticles exhibited more pronounced morphological changes, including improved surface integrity and cuticle fullness, compared with those observed on Day 7 (Figure 8A,B). Quantitative analysis of hair diameter (Figure 8C) further demonstrated the effects of AEBI treatment. Hair diameter increased with increasing AEBI concentration and treatment duration. In both the 7-day and 14-day cohorts, hair diameter gradually increased with increasing AEBI concentrations, and all AEBI-treated groups showed statistically significant differences compared with the blank control group. At equivalent AEBI concentrations, the 14-day groups exhibited markedly greater hair diameters than the corresponding 7-day groups. These findings indicate that AEBI treatment was associated with concentration- and duration-dependent changes in hair diameter and microscopic hair morphology. Higher AEBI concentrations and longer treatment durations were associated with more pronounced changes in these parameters. Together with the macroscopic morphological observations in Figure 8A and the quantitative analysis in Figure 8B, these findings further support the effect of AEBI treatment on hair coverage in the treated skin area [22,23].

## 3. Discussion

In recent years, driven by squid products, the squid processing industry has achieved rapid development. According to statistical data from the “2024 China Fisheries Statistical Yearbook”, China’s squid catch reached 314,600 tons, showing a sustained growth trend [24]. Currently, squid products have become a vital component of China’s aquatic processing industry [25,26,27]. During squid processing, by-products are generated, including internal organs, skin, ink, cartilage, and other residual tissues [1]. A few bioactive compounds have been isolated from cephalopod tissues such as eyes, skin, eggs, and other tissues of amphipods, and other components, for example, dihydroxanthommatin, Tyr-Ala-Ile-Val-Ala-Arg-Pro-Arg-Phe-NH2, SIP, and 4-Mercapto-1-methylhistidine. These compounds mainly consist of nitrogen-containing fused heterocyclic derivatives, other heterocyclic compounds, and polypeptides, which exhibit antioxidant and neuroprotective properties [28,29,30]. Several of these compounds have been successfully obtained through total chemical synthesis [28,29,30]. The ink sac small-molecule chemical constituents remain in the preliminary research stages.

Squid ink represents a rich source of bioactive compounds, including polysaccharides, melanin, and indole derivatives. It possesses well-documented antioxidant, anti-inflammatory, and tissue repair capacities. Specifically, squid ink melanin, which consists of indole monomers including 5,6-dihydroxyindole (DHI) and 5,6-dihydroxyindole-2-carboxylic acid (DHICA), can modulate cell signaling pathways, cell survival, and proliferation. For instance, melatonin, with antioxidant and vasodilatory properties, can facilitate hair regeneration. A key finding of the present study is that a novel quinolin molecule, AEBI (C_18_H_14_N_2_O_2_), was isolated from squid ink using enzymatic hydrolysis, chloroform extraction, Sephadex G-25 gel filtration, and ultrafiltration. Quinolin units are well-established modulators of multiple cellular signaling cascades. These molecules have been increasingly acknowledged for their hair growth–promoting potential.

Hair loss has become a prevalent cutaneous disorder affecting individuals of all genders, driving increasing research interest in natural hair growth–promoting ingredients as synthetic hair-regulating agents such as minoxidil. However, minoxidil is associated with adverse reactions including scalp irritation and systemic side effects. Against this background, the present study aimed to explore the hair growth–promoting activity of AEBI.

HFSCs are pivotal for sustaining the normal hair growth cycle and repairing impaired hair follicles [31,32]. AEBI exhibited the strongest efficacy in enhancing hair follicle stem cell (HFSCs) viability, exerting a proliferative effect. Its cellular activity reached nearly 200% at a concentration of 500 μg/mL. Our experimental data verify that the hair growth–promoting capacity of AEBI is mediated via multiple regulatory mechanisms: AEBI markedly enhances HFSC viability, an essential prerequisite for hair follicle regeneration. AEBI upregulates the expression of key functional proteins, including p63 (a critical marker for stemness maintenance), laminin and fibronectin (extracellular matrix proteins essential for hair follicle structural stability), as well as TGF-β (a vital growth factor governing cell proliferation and differentiation) [33].

Consistent with previous reports that squid ink extracts regulate cell proliferation-related proteins, the present study further identified AEBI as the core functional component responsible for hair growth modulation. Emerging evidence has highlighted its promising potential for hair care; squid ink extracts can penetrate hair follicles, repair damaged proteins, and remodel the scalp microenvironment.

The Wnt/β-catenin signaling pathway is a core cascade that governs hair follicle development, HFSC proliferation, and hair cycle transition [20]. Among them, LEF1, β-catenin, and Wnt10b are the characteristic proteins promoting hair growth [34]. In vivo results demonstrated that AEBI upregulates the expression of LEF1, β-catenin, and Wnt10b in rat dorsal skin, facilitates β-catenin nuclear translocation, and elevates the level of the proliferation marker Ki-67. Unlike minoxidil (MNX), which functions primarily by dilating scalp blood vessels and extending the anagen phase, AEBI exerts more comprehensive modulation on hair follicle biological behaviors, including promoting follicle maturation, increasing hair diameter, and improving cuticle regularity. These beneficial effects are more pronounced in high-concentration AEBI groups (500, 1000 μg/mL) compared with the MNX group.

The discovery of AEBI fills a critical research gap regarding the bioactive constituents of squid ink. Although prior studies have confirmed the general hair-growth benefits of squid ink extracts and attributed these effects to the synergistic action of polysaccharides and melanin, the present study is the first to isolate and identify a quinolin derivative (AEBI) with bioactivity. This finding not only deepens the understanding of the structure-activity relationship of squid ink–derived molecules, but also provides a specific molecular candidate for the development of natural hair-care agents. AEBI possesses a quinolin-centered chemical scaffold, and its unique functional groups may facilitate interactions with cellular receptors. Accordingly, AEBI can serve as a promising structural template for further optimization to improve bioavailability and therapeutic efficacy.

Several limitations of the present study should be acknowledged. All experiments were performed using mouse hair follicle stem cells and rat models. Further clinical investigations are required to validate the safety and efficacy of AEBI in humans. The precise binding target of AEBI within the Wnt/β-catenin signaling pathway remains unclarified, which warrants further verification via molecular docking and assays. In addition, the pharmacokinetic characteristics of AEBI, including skin penetration efficiency and metabolic stability, remain to be systematically evaluated to support its future development as a topical therapeutic agent.

In summary, the present study validated the hair growth–promoting potential of squid ink extract. Building upon previous investigations, we identified AEBI as a novel bioactive compound and elucidated its multifaceted regulatory effects, including the promotion of HFSC proliferation and modulation of key functional proteins. In animal models, high-dose AEBI exhibited comparable or enhanced efficacy relative to 5%MNX, indicating its promising potential as a natural candidate for alopecia intervention. Future studies should focus on optimizing topical delivery formulations of AEBI, conducting clinical trials to evaluate its safety and efficacy in human subjects, and exploring potential synergistic effects between AEBI and other bioactive ingredients. The present work not only improves the comprehensive utilization value of squid processing by-products but also provides for the development of safe and effective natural hair growth–promoting agents.

## 4. Materials and Methods

### 4.1. Reagents and Consumables

Trypsin-EDTA was purchased from Gibco (Grand Island, NY, USA). Phosphate-buffered saline (PBS) was obtained from Servicebio Technology (Wuhan, Hubei, China). CCK-8 was obtained from AbMole BioScience (Houston, TX, USA). Biomaterial: ink sac of *Symlectoteuthis oualaniensis*, harvested from the Indian Ocean in 2021. Reagents: Analytical-grade anhydrous ethanol, methanol, chloroform, sulfuric acid. were acquired from Sinopharm Chemical Reagent Co., Ltd. (Shanghai, China).; deuterated reagents such as dimethyl sulfoxide and pyridine were purchased from Sigma-Aldrich (Saint Louis, MO, USA); Chromatography-grade methanol and acetonitrile were purchased from J.T. Baker (Phillipsburg, NJ, USA); papain (CAS No. 9001-73-4) and trypsin (S31655) were purchased from Shanghai Yuanye Biotechnology Co., Ltd. (Shanghai, China); distilled water and ultrapure water were prepared in-house using a Milli-Q purification system.

### 4.2. Separation and Purification of Squid Ink Components

Filter the squid ink through an 80-mesh gauze and sonicate the filtrate in an ice bath for 1 h to enhance the solubility of melanin and other organic components in the squid ink. First, trypsin was added and incubated in a thermostatic water bath at 37 °C for 3 h. Subsequently, papain was added and incubated in a thermostatic water bath at 55 °C for 3 h. Both were used at the same concentration of 1% (*m*/*v*), equivalent to 1 g per 100 mL [35]. Upon reaction termination, rapidly heat the mixture to 100 °C and maintain it for 30 min to terminate enzymatic activity. Centrifuge the mixture at 10,000 rpm at 4 °C using a refrigerated centrifuge for 30 min, retain the supernatant, and store the precipitate at -80 °C for subsequent experiments.

The squid ink filtrate was extracted three times with chloroform using an equal volume of chloroform, and all organic extracts were pooled. The aqueous layer was loaded onto a Sephadex G-25 gel column (Cytiva, Marlborough, MA, USA) and eluted with 10% ethanol. Based on the results of thin-layer chromatography and liquid chromatography-mass spectrometry (6224 TOF MS) analysis, various fractions of the eluate were collected. The crude extract was then separated into fractions above 5 kDa and below 5 kDa using a Viva flow ultrafiltration module package.

Sample purification and repeated isolation were performed on a semi-preparative HPLC system: Shimadzu LC-20AT pump (Tokyo, Japan), SIL-20A autosampler, SPD-M20A PDA detector, and YMC-Pack ODS-AQ column (250 × 10 mm, particle size5 µm, 12 nm, flow rate 2.5 mL/min) for repeated preparation and purification of the samples.

### 4.3. Preparation and Cell Culture of HFSCs

Mouse hair follicle stem cells (HFSCs) and dedicated complete culture medium were purchased from Pricella Biotechnology Co., Ltd. (Nanjing, Jiangsu, China). The complete medium used for cell culture was supplemented with 10% fetal bovine serum (FBS) and 1% penicillin-streptomycin (P/S) mixed solution. HFSCs were routinely cultured and subcultured in a humidified incubator at 37 °C with 5% CO_2_, and the seventh-passage cells were adopted for all subsequent experiments to ensure cell activity and phenotypic stability. For the CCK-8 viability assay, HFSCs were seeded into 96-well plates (Corning Inc., New York, NY, USA) at a density of 1 × 10^4^ cells per well. For the enzyme-linked immunosorbent assay (ELISA), cells were plated in 12-well plates from the same manufacturer (Corning Inc., New York, NY, USA) at a density of 1 × 10^5^ cells per well.

### 4.4. CCK-8 Assay

100 μL of HFSCs suspension with a density of 1 × 10^4^ cells per well was seeded into 96-well plates, then incubated at 37 °C with 5% CO_2_. Cells were divided into the following groups: blank control (culture medium only), positive control (5% minoxidil at a final concentration of 10 μg/mL, based on preliminary dose-range tests), and AEBI treatment groups at final concentrations of 10, 50, 100, 500, and 1000 μg/mL. The experimental unit was the individual well; each group was set up with six replicate wells, and the entire experiment was independently repeated three times (n = 3). After respective treatments, 10 μL of CCK-8 reagent was added to each well, and the plates were incubated at 37 °C for 2 h. Finally, the absorbance of each well was measured at 450 nm using a microplate reader manufactured by Agilent (Santa Clara, CA, USA).

### 4.5. Preparation of Rat Depilation Model

Sprague–Dawley (SD) rats were used in this study. All animal procedures were reviewed and approved by the Institutional Animal Care and Use Committee of Shanghai Ocean University (Approval No. SHOU-DW-2023-220). All animal experiments were conducted in accordance with the institutional guidelines and ethical standards for laboratory animal care and use. The dorsal hair of each rat was shaved using a depilatory scalpel, followed by the application of depilatory cream to remove residual hair. After a 24-h recovery period, the dorsal skin of each rat was divided into four separate treatment regions. Each region received a different treatment. Seven treatment groups were included: AEBI at concentrations of 0, 10, 50, 100, 500, and 1000 μg/mL, and 5% minoxidil (MNX, 50 mg/mL) as the positive control. At each sampling time point, six rats were used, with each treatment represented in three different rats. Three rats contained four treatment regions, while the remaining three rats contained three treatment regions. Animals were divided into two groups for sample collection on Day 7 and Day 14, respectively. Therefore, a total of 12 rats were used. All treatments were applied topically once daily for 14 consecutive days. For topical administration, approximately 2–3 drops (approximately 100 μL) of each test solution were applied to the corresponding shaved dorsal region (2 × 2 cm) using a disposable plastic applicator tip, and then gently spread manually to ensure uniform coverage without damaging the skin. The solution was allowed to air-dry for approximately 2–3 min before the animal was returned to its cage [22,23,36,37].

### 4.6. Histological Observation of Hair Follicles (HE Staining)

After the experiment, rat dorsal skin tissues were processed following standard procedures. The tissues were first fixed overnight in 10% formalin solution at 4 °C, gradually dehydrated using graded ethanol, and then embedded in paraffin. Sections of 3 μm thickness were sliced with a pathological microtome and mounted on adhesive-coated glass slides for paraffin specimens. Subsequent staining was performed strictly according to standard SOPs. Sections were dewaxed in Xylene I and Xylene II for 20 min apiece, then rehydrated sequentially in absolute ethanol I, absolute ethanol II, and 75% ethanol for 5 min each. After thorough rinsing with tap water, hematoxylin-eosin (HE) staining was carried out. Stained sections were observed under an upright optical microscope (Olympus, Tokyo, Japan), and images were acquired via Slide Viewer software v2.6.0 (3DHISTECH Ltd., Budapest, Hungary). For each sample, three regions with the densest hair follicle distribution were selected and imaged at 100× magnification, followed by hair follicle quantification [17,19,20].

### 4.7. Enzyme-Linked Immunosorbent Assay (ELISA)

Hair follicle stem cells (HFSCs) were seeded into 12-well plates (Corning Inc., New York, NY, USA) at a density of 1 × 10^5^ cells/well. After 24 h of routine culture, the spent medium was discarded, and cells were rinsed with phosphate-buffered saline (PBS). In the experimental groups, the blank group received no treatment, whereas all other groups were treated with AEBI for 8 h. Subsequently, the residual medium was washed away using pre-cooled PBS, and cell lysis buffer was added to each well. After incubation on ice for 15 min, cells were harvested with a cell scraper. The cell suspension was centrifuged at 12,000 rpm at 4 °C for 10 min. The supernatant was harvested and stored at −80 °C for subsequent experiments.

According to the manufacturer’s instructions for the ELISA kit (Wuxi, Jiangsu, China), the expression levels of p63, laminin, fibronectin, and TGF-β were determined. The diluted cell supernatant and standard solutions were separately loaded into pre-coated ELISA plates and incubated at 37 °C for another 30 min. After five additional washes, the enzyme conjugate was added, and the mixture was incubated at 37 °C for another 30 min. After five additional washes, chromogenic solutions A and B were added sequentially. Following 30 min of light-shielded incubation at 37 °C, stop solution was promptly supplemented to terminate the reaction. Optical density of each well was measured at a wavelength of 450 nm using a microplate reader. All ELISA data were presented as the mean ± standard deviation (SD) from three independent experiments.

Skin tissues were harvested from rats in each group, trimmed, and accurately weighed. Pre-chilled homogenization buffer was added at a tissue-to-buffer mass ratio of 1:9 (900 μL buffer per 100 mg tissue). Samples were fully homogenized in an ice bath until a uniform homogenate was acquired. The homogenate was centrifuged at 3000–4000 rpm for 10 min at 4 °C. The supernatant was collected to prepare 10% tissue homogenate and temporarily preserved at 4 °C for subsequent detection. In accordance with the manufacturer’s guidelines of the ELISA kit (Wuxi, Jiangsu, China), the harvested tissue supernatant was used to measure the expression levels of LEF1, β-catenin, and Wnt10b.

### 4.8. Immunohistochemistry

Skin tissue samples from rats in each group were collected, followed by fixation, dehydration, and embedding to prepare paraffin sections. The sections were sequentially immersed in environmentally friendly dewaxing solution I, II, and III for 10 min each, followed by immersion in absolute ethanol I, II, and III for 5 min each, and rinsed with distilled water to complete dewaxing and rehydration. Appropriate antigen retrieval solution (pH 6.0 citric acid or pH 8.0/9.0 Tris-EDTA buffer) was selected based on tissue type for retrieval, with precautions taken to prevent section drying. After natural cooling to room temperature, the sections were washed three times for 5 min per wash with pH 7.4 PBS buffer on a decolorizing shaker. Subsequently, the sections were incubated in 3% hydrogen peroxide solution at room temperature in the dark for 25 min to block endogenous peroxidase activity. After PBS rinsing, 3% BSA (normal rabbit serum was used for goat-derived primary antibodies) was added dropwise for non-specific blocking at room temperature for 30 min. The blocking solution was discarded, and PBS-diluted primary antibodies were applied to the sections, followed by overnight incubation at 4 °C in a humidified chamber. The next day, after three PBS washes, HRP-conjugated secondary antibodies matching the host species of the primary antibody were added and incubated at room temperature for 50 min. Following r three additional PBS washes, freshly prepared DAB chromogenic solution was added. The chromogenic reaction was monitored microscopically, and the reaction was terminated by tap water rinsing once brownish-yellow positive signals were observed. The sections were counterstained with hematoxylin for 3 min, differentiated with differentiation solution for several seconds, and blued with bluing solution. After gradient dehydration (75% ethanol → 85% ethanol → absolute ethanol → n-butanol) and xylene clearing, the sections were mounted with mounting medium and observed under a bright-field microscope. Cell nuclei were stained blue, and positive expression of the target protein was presented as brownish-yellow staining [38].

### 4.9. Scanning Electron Microscope (SEM)

For each group, small hair samples harvested from the dorsal skin were directly affixed to conductive adhesive. Only guard hairs (the longest and thickest hairs on the dorsal surface) were selected for measurement, while zigzag/undercoat hairs were excluded to ensure consistency of hair type across all groups. At least 20 guard hairs were randomly collected from each treatment region for diameter measurement. Gold sputtering was subsequently performed for 45 s at a sputtering current of 10 mA using a Quorum SC7620 sputter coater (Quorum Technologies, East Sussex, UK). Subsequently, a ZEISS Sigma 300 scanning electron microscope (SEM) (Carl Zeiss AG, Oberkochen, Germany) was used for sample morphological observation and energy-dispersive spectroscopy (EDS) elemental mapping. For morphological imaging, the accelerating voltage was set to 3 kV. For EDS mapping analysis, the accelerating voltage was adjusted to 15 kV, and an SE2 secondary electron detector was applied for image acquisition.

### 4.10. Statistical Analysis

Data are presented as mean ± standard deviation (SD). One-way analysis of variance (ANOVA) or independent *t*-test was used for statistical analysis. Compared with the control group, * *p* < 0.05, ** *p* < 0.01, *** *p* < 0.001 indicate statistically significant differences. All statistical analyses were performed using SPSS 27.0 software, and all graphs were plotted using GraphPad Prism software 10.0.

## Figures and Tables

**Table 1:** NMR spectra data of AEBI.

No.	^13^C (ppm)	^1^H (ppm)	No.	^13^C (ppm)	^1^H (ppm)
1	127.2		1’	127.25	
2	141.34		2’	141.28	
3	119.58	8.08 (*d*, J = 8.0 Hz, 1H),	3’	118.74	7.71–7.65 (*m*, H),
4	123.7	7.41 (*t*, J = 7.4 Hz, 1H),	4’	131.95	7.59 (*t*, J = 7.6 Hz, 1H),
5	126.17	8.85–8.80 (*m*, H),	5’	123.26	7.37 (*t*, J = 7.5 Hz, 1H),
6	166.15		6’	126.17	8.85–8.80 (*m*, H),
7	111.24	7.71–7.65 (*m*, H),	7’	139.64	7.98 (*d*, J = 7.3 Hz, 1H),
8	132.61	7.71–7.65 (*m*, H),	8’	109.95	6.60 (*d*, J = 7.2 Hz, 1H),
9	179.62		9’	178.54	
1-NH			9’-NH2		

## Data Availability

The datasets generated and/or analyzed during the current study are not publicly available due to the requirements of the project but are available from the corresponding author on reasonable request.

## Supplementary Materials

The following supporting information can be downloaded at: https://www.mdpi.com/article/10.3390/ijms27209039/s1.
