# Supplementary material for: A Novel Squid Ink–Quinolin Derived Compound (AEBI), Promotes Hair Growth and Enhances Hair Follicle Stem Cell Activity
Source: Int J Mol Sci. 2026 Oct 12;27(20):9039. doi: 10.3390/ijms27209039 (PMC13705025; doi:10.3390/ijms27209039)
Supplement: Supplementary file 1 [file ijms-27-09039-s001.zip › ijms-4537631-supplementary.pdf]

Supplementary Materials

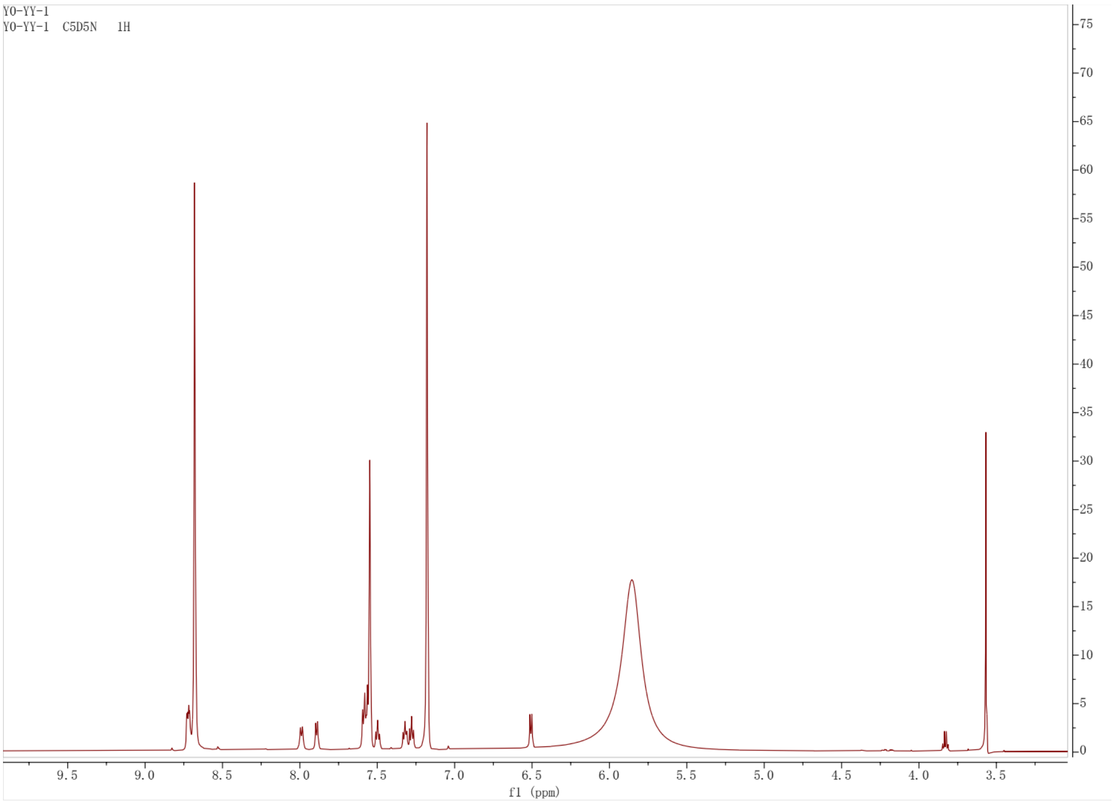

Figure S1. ^{1}H-NMR Spectrum of AEBI

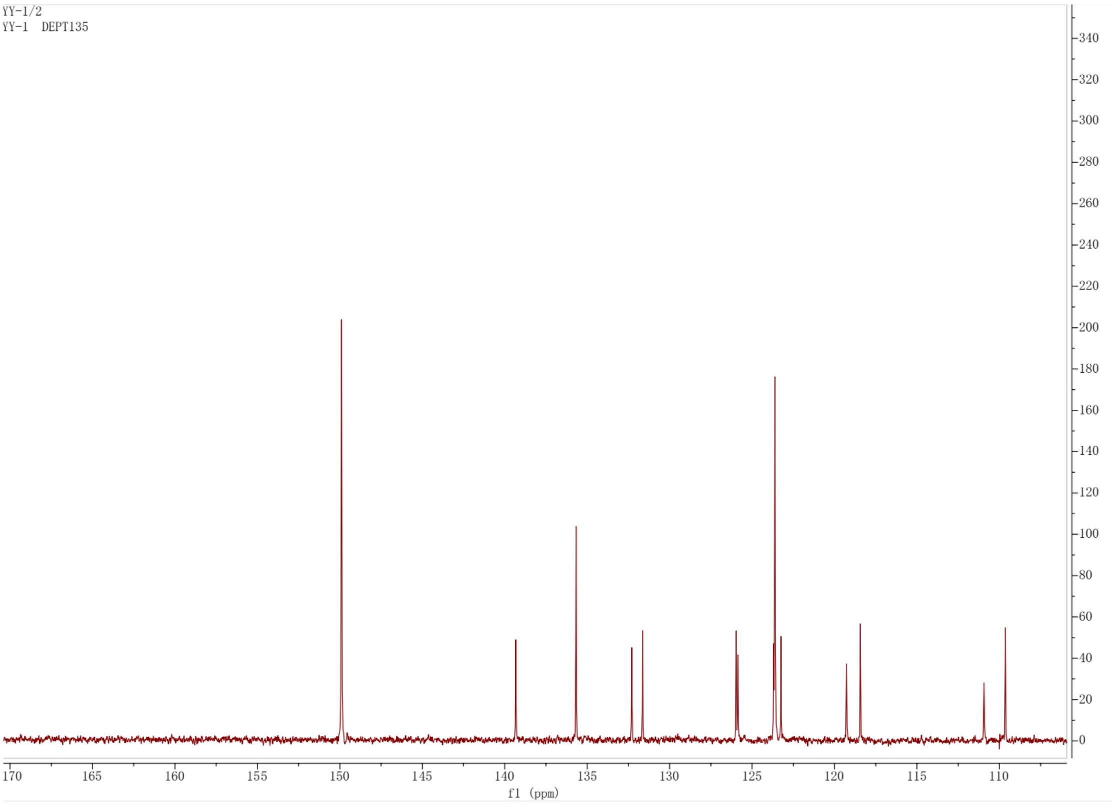

Figure S2. ^{13}C-NMR Spectrum of AEBI

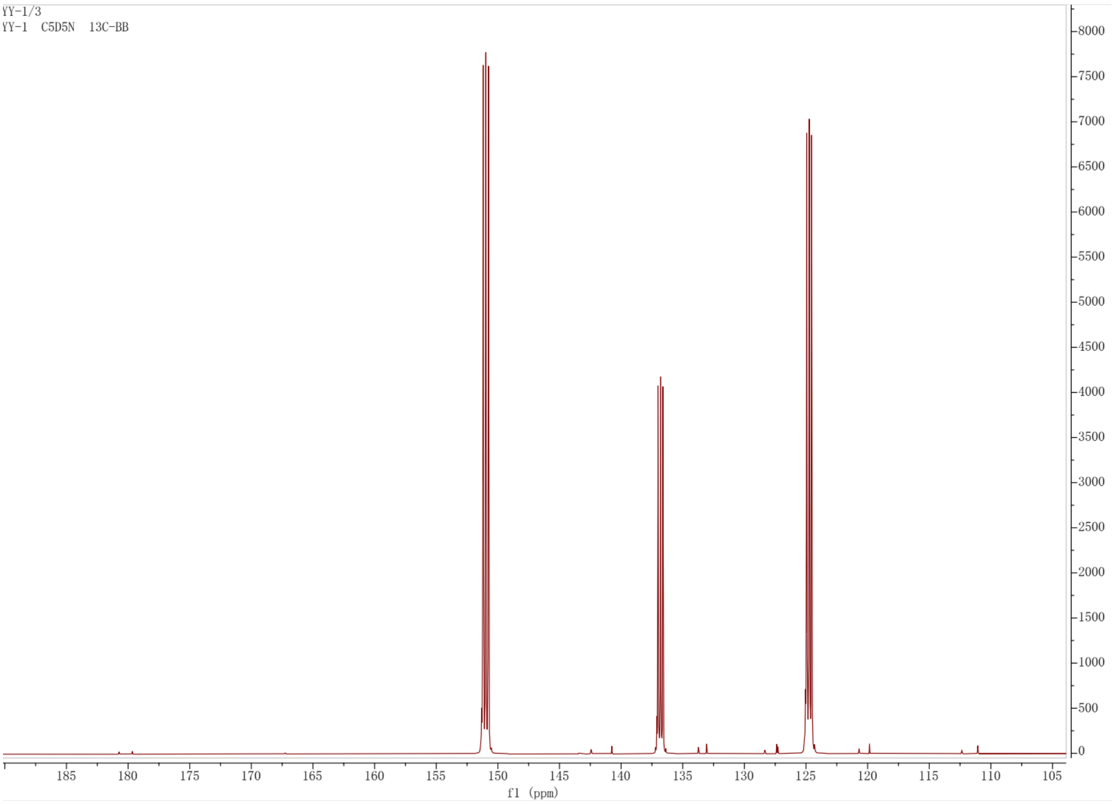

Figure S3. ^{13}C-NMR Spectrum of AEBI

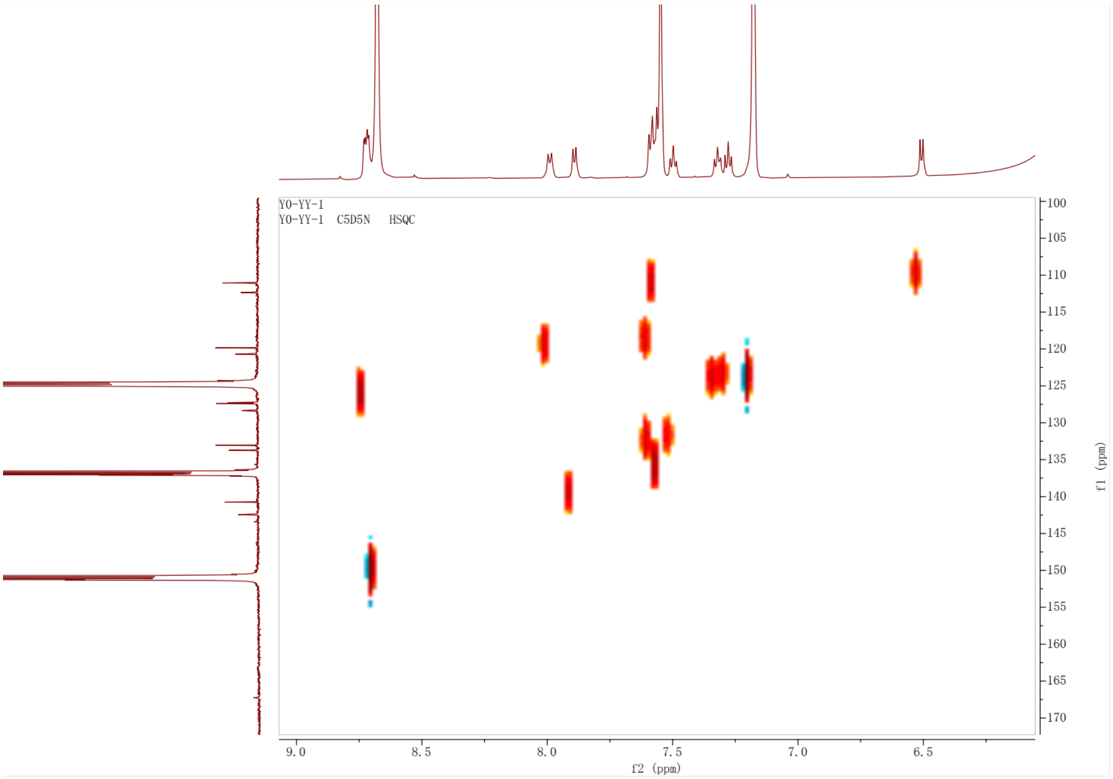

Figure S4. HSQC Spectrum of AEBI

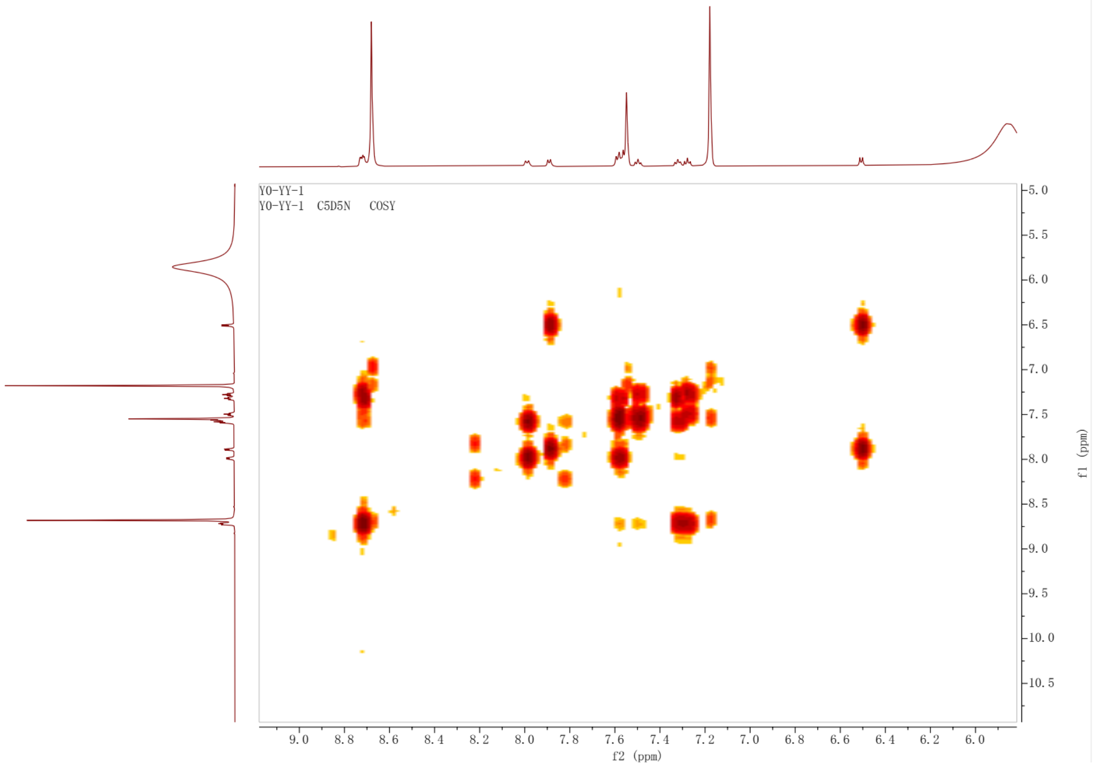

Figure S5. ^{1}H-^{1}H COSY Spectrum of AEBI

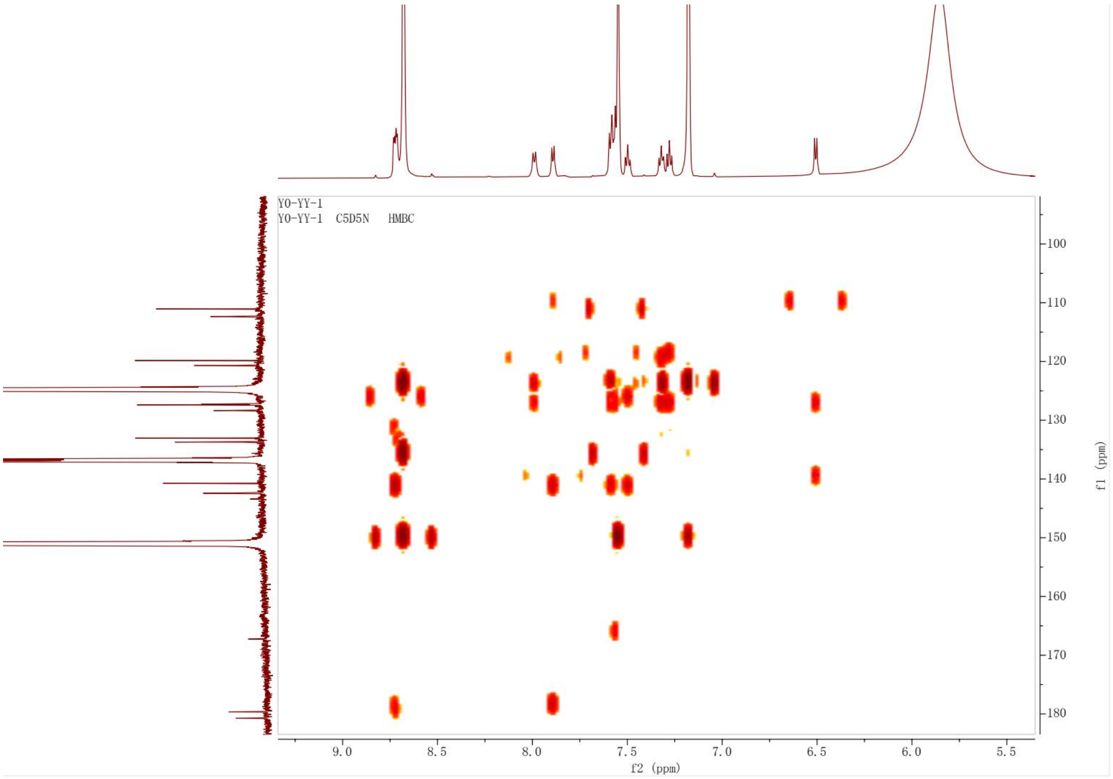

Figure S6. HMBC Spectrum of AEBI

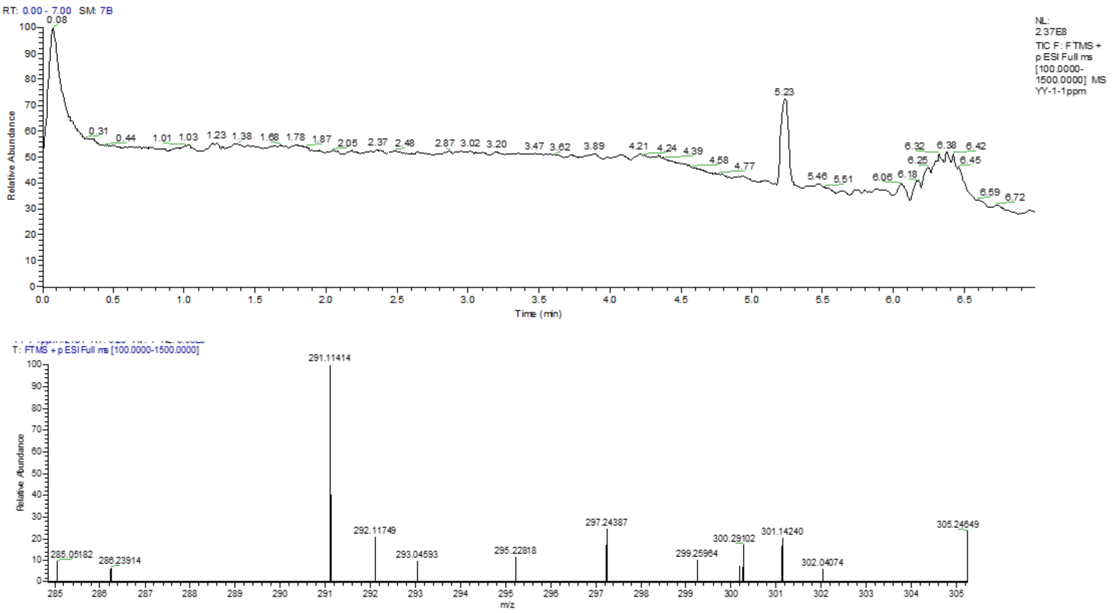

Figure S7. HRESIMS Spectrum of AEBI
